# A Practical Treatise on the Diseases Peculiar to Women

**Published:** 1843-10

**Authors:** 


Art. IX —*A Practical Treatise on the Diseases peculiar to Women.* By Samuel Ashwell, m.d. Part II, Organic Diseases.—*London,* 1843. 8vo, pp. 260.

We noticed the first part of this work in our Twenty-first Number, (Jan. 1841,) and gave a very favorable report of its general character and contents. Like almost all authors who allow themselves to be seduced into the publication of their works in parts, Dr. Ashwell has found—and his readers to their cost have found—that performance lags sadly behind promise in such cases. In September, 1840, we were told by Dr. Ashwell in Part I, that Part II would appear in December of the same year, while Part III, completing the work, would be published in March, 1841. Such is the promise: now for the performance: Part II appears in the summer of 1843, and announces that Part III is nearly ready! We believe Dr. Ashwell has better reason than many authors for pleading the old excuse of want of time; still we do not acquit him of blame for having consented to allow of the publication of a small portion of a work, bearing the specific promise of speedy completion, while the remainder was yet unwritten, and subject to all the accidents of a busy profession and uncertain life. We trust that the promise now given will be better kept. We shall wait until we see that it is so, before we give any detailed account of the present part. We will merely inform our readers that, besides chapters on hysteria and irritable uterus, it treats, in succession, of the following subjects: Tumours of the uterus; Premature labour complicated with organic disease; Organic diseases of the os and cervix; Congestion of the uterus; Metritis; Cancer; Ulcer and excrescence of the uterus; Rigidity and occlusion of the cervix; Diseases of the mucous membrane and polypus of the uterus. We may add, in conclusion, that the present part bears out the character we gave of the former. There is nothing very novel or brilliant in it; but it gives a good account of the different diseases treated of, and contains many plain, practical directions which we know not where to look for elsewhere under one head. To young practitioners, in particular, Dr. Ashwell's book cannot fail to be very useful.

